# Does function fit structure? A ground truth for non-invasive neuroimaging

**DOI:** 10.1016/j.neuroimage.2014.02.033

**Published:** 2014-07-01

**Authors:** Claire Stevenson, Matthew Brookes, José David López, Luzia Troebinger, Jeremie Mattout, William Penny, Peter Morris, Arjan Hillebrand, Richard Henson, Gareth Barnes

**Affiliations:** a School of Physics and Astronomy, Nottingam, UK; b SISTEMIC, Engineering Faculty, Universidad de Antioquia, Medellín, Colombia; c Wellcome Trust Centre for Neuroimaging, London, UK; d INSERM, Lyon, France; e VU University Medical Center, Amsterdam, The Netherlands; f MRC CBU, Cambridge, UK

## Abstract

There are now a number of non-invasive methods to image human brain function in-vivo. However, the accuracy of these images remains unknown and can currently only be estimated through the use of invasive recordings to generate a functional ground truth. Neuronal activity follows grey matter structure and accurate estimates of neuronal activity will have stronger support from accurate generative models of anatomy. Here we introduce a general framework that, for the first time, enables the spatial distortion of a functional brain image to be estimated empirically. We use a spherical harmonic decomposition to modulate each cortical hemisphere from its original form towards progressively simpler structures, ending in an ellipsoid. Functional estimates that are not supported by the simpler cortical structures have less inherent spatial distortion. This method allows us to compare directly between magnetoencephalography (MEG) source reconstructions based upon different assumption sets *without* recourse to functional ground truth.

## Highlights

• We use spherical harmonics to create generative cortical surface models.
• Accurate functional estimates will be best supported by veridical cortical models.
• The method provides spatial confidence bounds for non-invasive functional images.

## Introduction

Functional neuroimaging aims to non-invasively image the spatial, temporal and in some cases spectral signature of human brain function *in vivo*. Methods include electroencephalography (EEG) and magnetoencephalography (MEG), which measure electric and magnetic fields induced directly by electrical current flow in neuronal assemblies; and positron emission tomography (PET) and functional magnetic resonance imaging (fMRI), which image brain function indirectly via induced metabolic changes. However, the spatial distortion of functional images can be questioned since the ground truth (i.e. which brain areas are truly exhibiting functional change) is always unknown (even invasive electrode recordings can only provide a window on a small area of brain tissue and have an imperfectly characterised sensitivity to other regions). The question of spatial distortion is a problem for all neuroimaging modalities, but is particularly important in MEG and EEG since measured data must be converted from magnetic or electric fields measured outside the head to current flow estimates in the brain. This is an ill-posed inverse problem and additional prior information, or an underlying model of neural activity, is required to solve it.

**Fig. 1:**
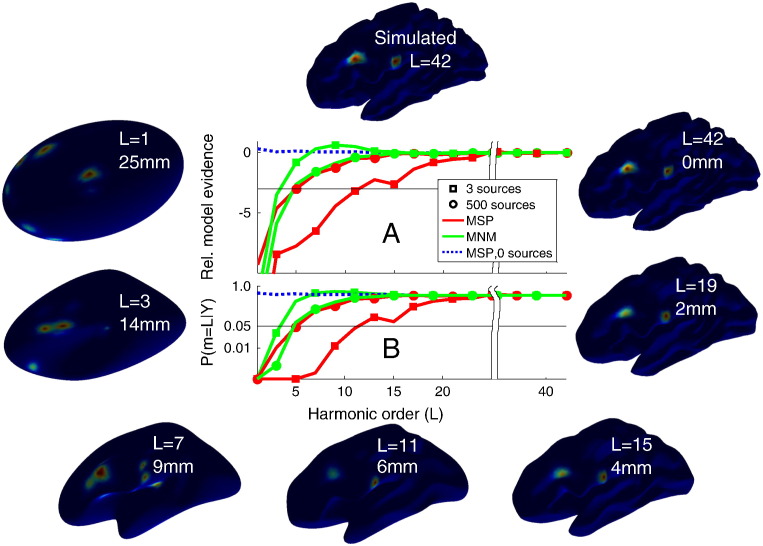
The figure border shows the MSP reconstructed current density maps of simulated data onto progressively simpler (clockwise) cortical structures. Sources were simulated on the full cortical surface model (top panel, 2 sources visible); these data were then reconstructed using either MSP or MNM algorithms onto surface models of progressively simpler harmonic structure (with *L* indicating harmonic order alongside the 95th percentiles of spatial distortion from surface *L* = 42). Panel A shows the difference in log model evidence between a reconstruction of the original data onto the true cortical surface and reconstructions onto simpler surfaces. Panel B shows the fixed effects probability that a lower harmonic model improves upon the complete cortical model (*L* = 42). The original data consisted of either 3 simulated sources with FWHM ~ 10 mm (shown by squares); 500 simulated point sources (shown by circles) or no simulated sources (dotted). The reconstructions using MSP and MNM are denoted by red and green coloured lines respectively. For the MSP reconstruction of the 3 source data (red squares), it is clear that the simulated MEG data are very unlikely to be explained by a cortical model of low harmonic order; as the harmonic order increases the solution improves until it reaches a point where it cannot be distinguished from the full model. The point at which we can discriminate between good and bad cortical models (curves cross *p* < 0.05 line in panel B) gives the highest distinguishable harmonic (HDH) model order, a lower bound on the accuracy of the functional estimate (in this case around HDH = 11). In contrast to MSP, the MNM assumptions for these data (green squares) barely distinguish between cortical models (HDH = 3). If we use simulated data closer to the minimum norm assumptions (a large number of uncorrelated point sources, shown by circles), the sensitivity of the MSP inversion to the cortical surface degrades (red circles) whereas the MNM algorithm (green circles) improves. The blue curve shows the (MSP reconstructed) noise-only case, demonstrating that no cortical model is any worse than the true model (in the absence of useful functional data).

Here we introduce a general framework that enables the spatial distortion of a functional brain image to be estimated empirically. The principal idea is that we know brain function, as measured by all of the above techniques, is localised within anatomically-identifiable grey matter structure. If we make a generative model based on grey matter structure, we can test how sensitive our functional estimate is to changes in the anatomical information underlying the model. If the functional estimates are veridical then an accurate anatomical model will be required to support them. Conversely, if the functional data are inaccurate or imprecise, then better anatomical models will have little advantage over poorer ones. Recently, by translating and rotating the cortical manifold we showed how the evidence for such cortical generative models was a monotonic function of accuracy (Lopez et al., 2012b). We now use a similar approach but work with Fourier representations of these surfaces. We create cortical surfaces which all have the same mean location but differ in their spatial frequency content. All models have the same number of vertices and topology but the spatial frequency content is determined by the number of spherical harmonic components used to describe the surface (Fig. 1). At each harmonic order, we can quantify the spatial distortion from the true anatomy; in this case, we used the 95th percentile of the distribution of distance errors to the true anatomy (shown alongside the harmonic order in Fig. 1) as a measure of spatial distortion. The higher the harmonic order, the smaller the spatial distortion of the cortical model from the true anatomy. We use these cortical surfaces as generative models of MEG data, which we know to derive primarily from dendritic current flow within pyramidal neurons oriented normal to the cortical sheet (Okada et al., 1997). A Bayesian statistical framework allows us to compute the model evidence for optimised current flow estimates for each of these anatomical models. We compute the evidence for progressively lower harmonic surface models (simpler anatomical structures) until we arrive at one that does not support the functional data. We call this the highest distinguishable harmonic (HDH) surface model. The HDH model gives an upper bound on the spatial distortion (or a lower bound on the accuracy) we can expect in the functional image. For example, if functional imaging data were due to noise (rather than neuronal activity), one would expect the evidence for a cortical surface shaped like a brain to be similar to that for a brain shaped like a rugby ball. However, if the functional data can be explained by cortical current flow, then more anatomically accurate models should have higher evidence. The main advantage of this approach is that no *a priori* knowledge is required of where the activity should be; the only assumption is that current flow should originate in the grey matter.

## Methods

We first explain the construction of the different cortical manifolds used and then go on to describe the different inversion schemes.

## Spherical harmonics

We computed a weighted Fourier series (WFS) representation of the canonical cortical mesh (Mattout et al., 2007) allowing this surface to be expressed as a weighted linear combination of spherical harmonics (Chung et al., 2007). The WFS can be expressed as a kernel smoothing technique described by

(1) $$F_{\sigma }^{k}\left[f\right]\left(\omega \right)=\sum_{l=0}^{L}\sum_{m=-l}^{l}e^{-l\left(l+1\right)\sigma }f_{\mathrm{lm}}S_{\mathrm{lm}}\left(\omega \right)$$

where *σ* is the bandwidth of the smoothing kernel, *L* is the harmonic order of the surface, *S_lm_* is the spherical harmonic of degree *l* and order *m*, and the Fourier coefficients are given by *f*_*lm*_ = 〈*f*, *S*_*lm*_〉, where *f* is determined by solving a system of linear equations (Chung et al., 2007). *ω* is the spherical parameterisation of a unit sphere, given in terms of the polar angle *θ* and azimuthal angle *φ* as

(2) $$\omega =\left(\sin\theta \cos\phi ,\sin\theta \sin\phi ,\cos\theta \right)$$

with *ω* = (*θ*, *ϕ*) ∈ [0, *π*] ⊗ [0, 2*π*].

We looked at harmonic series ranging from *L* = 1 to 42 (all other parameters as in (Chung et al., 2007)). Each surface has the same number of vertices (*N*_*d*_ = 8192) and topology.

## Spatial distortion

For each harmonic order *L* we computed a vector $d_{L}\in {ℜ}^{1\times N_{d}}$ of per vertex distortions (in mm) with respect to the most comprehensive harmonic representation (*L*_max_):

(3) $$d_{L}=\sqrt{\left(x_{L}-x_{L\max}\right)\cdot \left(x_{L}-x_{L\max}\right)}$$

Where ⋅ is the dot product operator, $x_{L}\in {ℜ}^{3\times N_{d}}$ are the coordinates of the *N*_*d*_ vertices in the reduced harmonic form and $x_{L\max}\in {ℜ}^{3\times N_{d}}$ are the *N*_*d*_ vertices of the most complete harmonic representation (*L* = 42 in this case). In this manuscript we define spatial distortion for harmonic surface *L* to be the 95th percentile of the per vertex distortions in **d**_*L*_.

## Source reconstruction

The MEG/EEG inverse problem can be expressed concisely within a Bayesian framework in which prior assumptions made about source covariance differentiate between most popular inversion algorithms (Wipf and Nagarajan, 2009). In this work, we use a Parametric Empirical Bayesian (PEB) framework (Henson et al., 2011, Mattout et al., 2006, Phillips et al., 2005) that allows us to switch between different functional and anatomical inversion assumptions. Here, we used the framework outlined in (Friston et al., 2008b) for source reconstruction. The algorithm provides a generic framework to optimally weight and select between a candidate set of covariance matrices: In brief, the MEG/EEG data can be related to the neural activity that generates it using the linear model:

(4) Y=KJ+ϵ

where $Y\in {ℜ}^{N_{c}\times N_{t}}$ is the sensor data, where *N*_*c*_ = 274 is the number of sensors (normally 275 but one channel turned off) and *N*_*t*_ is the number of time samples; $K\in {ℜ}^{N_{c}\times N_{d}}$ is the lead field matrix that maps the *N*_*d*_ source locations to the *N*_*c*_ channels; $J\in {ℜ}^{N_{d}\times N_{t}}$ is the current distribution at each source location; and ϵ is zero mean Gaussian noise. We used a single shell (Nolte, 2003) based on the inner surface of the skull to define the forward model.

In practice it is convenient to reduce the dimensionality of the problem by taking the dominant eigenmodes of both the lead field matrix and the data. In this manuscript we used 100 spatial and 16 temporal modes. For clarity of notation we omit this stage here and continue with *N*_*c*_ channels and *N*_*t*_ samples, but see Friston et al. (2008b) and Lopez et al., 2012b, Lopez et al., 2014 for a complete description.

Under Gaussian assumptions, the solution to Eq. (4) can be expressed as the maximisation problem:

(5) $$\hat{J}=E\left[p\left(J|Y\right)\right]\propto \arg\max_{J}p\left(Y|J\right)p_{0}\left(J\right)$$

Where *E* denotes the expected value, the likelihood is $p\left(Y|J\right)=N\left(Y;\mathrm{KJ},\Sigma_{\epsilon }\right)$ and the prior probability distribution is $p_{0}\left(J\right)=N\left(J;0,Q\right)$, assuming a priori that **J** and **ϵ** are zero mean Gaussian processes with covariances ***Q*** and **Σ**_ϵ_ respectively, and N is the multivariate normal probability density function.

If the source covariance, ***Q***, is known then source activity $\hat{J}$ can be estimated directly (Friston et al., 2008b)

(6) $$\hat{J}=QK^{T}{\left(\Sigma_{\epsilon }+\mathrm{KQ}K^{T}\right)}^{-1}Y$$

Where *^T^* denotes a matrix transpose. Here we assume that sensor noise **Σ**_*ϵ*_ = *h*_0_***I***_*Nc*_ is independent and uniformly distributed, with $I_{N_{c}\;}$ an (*N*_*c*_ × *N*_*c*_) identity matrix and *h*_0_ a hyperparameter effectively controlling the regularisation. Different M/EEG algorithms entail different choices of the prior source covariance ***Q*** (Friston et al., 2008b, Wipf et al., 2010). For the minimum norm (MNM) solution, ***Q*** is simply an (*N*_*d*_ × *N*_*d*_) identity matrix; for the Multiple Sparse Prior (MSP) solution, ***Q*** comprises an optimised mixture of a library of *N*_*q*_ covariance components $C=\left\{C_{1},\ldots ,C_{N_{q}}\right\}$:

(7) $$Q=\sum_{i=1}^{N_{q}}h_{i}C_{i}$$

where here we use *N*_*q*_ = 512. Each component describes the covariance of a single connected patch of cortex (FWHM ~ 10 mm), weighted by the set of hyperparameters $h=\left\{h_{1},\ldots ,h_{N_{q}}\right\}$ (though other choices of sparse support are possible). The algorithm then uses a non-linear search to optimise the hyperparameters using the variational free energy as a cost function (Friston et al., 2008a). Briefly, the negative variational free energy is a trade-off between the accuracy of the model in explaining the data, and the complexity of achieving that accuracy (Penny et al., 2010):

(8) $$F\left(h\right)=\mathrm{accuracy}\left(Y,\hat{J}\left(h\right)\right)-\mathrm{complexity}\left(\hat{J}\left(h\right)\right)$$

This maximisation returns an approximate lower bound on the log model evidence $F\left(\hat{h}\right)\approx \log p\left(Y\right)$ (Friston et al., 2007). In the MSP case, where there are many hyperparameters, the optimization is achieved (here) using a Greedy Search algorithm (Friston et al., 2008a).

For simulated data, only covariance priors based on the initial MSP patch library were used (as the sources were simulated at these vertices). However for the reconstruction of empirical data (onto each surface mesh), as the patch centres are unknown a-priori, we inverted the same data 16 times, each time using a different randomly centred set of 512 patches (i.e. a different set of priors) and chose the solution with highest free energy (Lopez et al., 2012a, Troebinger et al., 2013).

In this study we compare cortical surface models in two ways. In order to get a robust differentiation between cortical models that do and do not support the data, we use a pairwise comparison between the true cortical anatomical model (made up of 42 harmonics here) and successively lower harmonic orders. Under flat priors (*p*(*m* = *L*) = 0.5 and *p*(*m* = 42) = 0.5) then

(9) $$p\left(m=L|Y\right)=\frac{p\left(Y|m=L\right)}{p\left(Y|m=L\right)+p\left(Y|m=42\right)}$$

This series of pairwise comparisons gives us the most complex harmonic model (the HDH) that is distinguishable from the true anatomy.

We are then left with a subset of anatomical models above the HDH that support the data and are not significantly different from the true anatomical model. In order to compute the relative probabilities of these models we used a fixed effects analysis (Stephan et al., 2009). Where under flat priors, the posterior probability of surface model *L* is given as

(10) $$p\left(m=L|Y\right)=\frac{p\left(Y|m=L\right)}{\sum_{m=\mathrm{HDH}+1}^{m=42}p\left(Y|m\right)}$$

Here, as we are interested in induced changes we show the joint posterior over anatomy and modulation in power on each cortical surface as a log power ratio (1 second pre-stimulus vs. 1 second post stimulus) at each vertex.

## Simulations

We used the 42 harmonic decomposition of a canonical (Mattout et al., 2007) cortical (grey-white matter boundary) mesh with 8192 vertices. Active cortex was simulated to best match either MSP or MNM prior assumptions. We used either 3 sources with the same smoothed impulse response as MSP (full width half maximum FWHM = 10 mm) or 500 sources with no spatial extent (to be most consistent with MNM priors). We included a condition in which the simulated sources had zero amplitude (i.e. purely sensor noise). We ran each scenario 16 times, with sources simulated at a random location drawn either from the MSP patch library (without replacement) for the MSP case, or randomly across the vertices for the MNM case. Each active source was given a white noise time course for 161 samples. For all simulations we used a single trial of data (sampled at 200 Hz) with an SNR of 0 dB, meaning that the average signal power (over channels) was equal to the sensor noise level. We then used either MNM or MSP priors to estimate the cortical current distribution (and associated log model evidence) on each of the harmonic surfaces.

## Data acquisition

For validation we used data from one healthy subject who carried out a visually cued, skilled right hand finger movement task. In each trial finger-thumb opposition was carried out for 16 s followed by 16 s of rest, with each experiment comprising 20 trials. All experiments were approved by the University of Nottingham Medical School Ethics Committee.

MEG data were recorded using a third order synthetic gradiometer configuration of a 275-channel CTF whole-head MEG scanner (one channel failed giving 274 useful channels), with a sampling rate of 600 Hz and hardware anti-aliasing filters at 0–150 Hz. Prior to MEG data acquisition, head localisation coils (HLCs) were placed in perspex mounts glued to the scalp at the nasion and pre-auricular points. HLCs were localised inside the scanner continuously during data acquisition with a motion tolerance of 5 mm enforced.

Following MEG data acquisition, MR visible markers were placed in the same perspex mounts and T_1_ weighted structural anatomical MR images acquired using a Philips 3 T MR Achieva System, scan parameters (TR = 8.1 ms, TE = 3.7 ms, flip angle = 8°, 256 × 256 × 168 matrix size). Co-registration to MEG measurement space was achieved by matching of MR visible markers to the HLC locations.

## Results: simulation

We consider two possible models of cortical current flow: (*i*) all current sources equally likely to be active but with minimal total energy (MNM); and (*ii*) the activity consists of a sparse set of active regions (MSP, see (Friston et al., 2008b)).

The topmost cortical surface in Fig. 1 shows an example of one simulated iteration of the 3 source scenario (2 of the 3 randomly selected locations are visible from this view). These simulated sources were used to generate MEG data (with signal-to-noise ratio (SNR) of 0 dB) that was subsequently reconstructed using different inversion assumptions. The surfaces around the edge of the figure show activity estimated according to the MSP reconstruction of the MEG data on progressively simpler cortical structures defined by the number of spherical harmonics (*L* = 1–42). It is apparent that when we try to reconstruct onto a simpler surfaces (e.g. *L* = 1), a more complex current distribution is required to explain the same data. Model evidence is a cost function that trades off accuracy of data fit against complexity (more active regions): the most likely estimates of current flow will therefore be the simplest ones that explain the most data. Panel A shows the average relative difference in (log) model evidence between the complete (*L* = 42) generative model and progressively simpler ones. Negative values at low harmonic orders mean that these surface models are less likely than the true cortical surface (a difference of 3 (thin solid line) equates to a model being 20 times less likely). For each surface, the relative model evidences of two current distributions are shown: one reconstructed using the MNM assumptions (green line) and the other using assumptions implicit in MSP (red line). Reconstructions of the 3 and 500 source scenarios are shown as squares and circles respectively. Panel 1B shows the probability (based on a series of pairwise comparisons, Eq. (9)) that a lower order surface would be a better model than the most complete version of the anatomy available (42 harmonics). Again the thin solid line shows the point at which a cortical model is twenty times less likely than the true model. The point at which each curve crosses this line gives the HDH model order (upper bound on the spatial distortion, or a lower bound on accuracy) of this functional estimate. It is clear that the MSP reconstructions of the 3 source scenario (red squares) are sensitive to surface structure and that model evidence increases monotonically with harmonic order; in this case it is possible to distinguish up to harmonic 11 (HDH = 11) from the true cortical surface. In contrast, the current distribution estimate for the 3 source data based on the MNM assumptions (green squares) has very little dependence on the anatomical generative model, and only differentiates between an almost ellipsoidal cortex (HDH = 3) and the true cortical surface. If however we look at the difference between MSP and MNM reconstructions of the 500 source scenario (red and green circles respectively) we see very similar performance (HDH around 5). Importantly, reconstructions of MEG data that are entirely due to noise (i.e. not due to cortical activity—labelled 0 sources in Fig. 1) cannot differentiate between anatomical models (MSP reconstructions of noise, blue dotted line).

## Results: experimental recordings

**Fig. 2:**
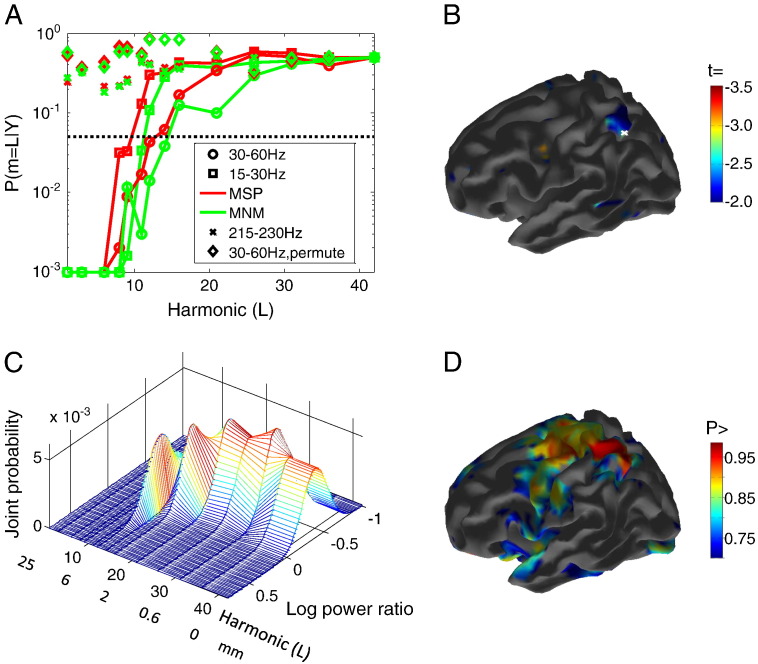
Reconstruction of cortical activity underlying a skilled finger movement task. Probability of lower harmonic cortical models supporting MSP (red) and MNM (green) functional estimates from MEG data during a complex finger movement task for 15–30 Hz (squares), 30–60 Hz (circles) and 215–230 Hz (crosses) frequency bands. In this case it is clear that both MSP and MNM assumptions are reasonable for both physiological bands because in all cases the functional estimates support more complex anatomy (higher order harmonics). For the non-physiological (215–230 Hz) case, by contrast, we cannot rule out even the lowest harmonic surfaces as possible models. For both physiological bands the MNM functional estimate is supported by higher accuracy anatomy. In the 15–30 Hz band, HDH = 9 for MSP and 11 for MNM, corresponding to spatial distortions of ± 7.2 and ± 6.0 mm respectively; for 30–60 Hz, HDH = 12 (± 5.4 mm) for MSP and 14 (± 4.2 mm) for MNM. As a further control we performed the same tests on the 30–60 Hz band data but randomly interchanged the MEG channel locations (destroying the link with underlying anatomy); again we see no dependence on cortical structure (diamonds). Panel B shows the MSP estimated *t*-statistic map of power change (1 s pre vs. 1 s post stimulus) in 15–30 Hz band power. The map is displayed on the cortical model (*L* = 31) with the highest posterior probability from the candidate models above the HDH threshold (models 10–42 in this case). Panel C shows joint distribution over beta (15–30 Hz) band modulation (as a log of the power ratio so that negative values mean power decrease) and cortical model. As the range of harmonic surfaces from *L* = 10 to *L* = 42 support these data equally well (the curve is not strongly peaked at any harmonic), we can say that the spatial error bounds on this estimate are around ± 6 mm. It is clear that the modulation estimate is dependent on the cortical model, with cortical models of around 10 harmonics equally likely to show power increases as power decreases. We can however calculate the probability that power decreased at this vertex by integrating all cortical models over the area under the curve for negative log modulation (*p* > 0.94 in this case). In Panel D, we show this integral, i.e., combined probability of a distortion less than 6 mm (*L* = 10) and a power decrease, across the whole cortical surface.

We applied the same method to look at data from an experimental MEG recording of a skilled finger movement task. In this case rather than pool model evidence values over simulations, we pooled over 2 s data segments from − 3 to + 3 s with respect to movement onset. Fig. 2A shows the probability that a lower harmonic order model performs as well as the full anatomical model for the two different inversion algorithms in two physiological frequency bands (15–30 Hz—beta, 30–60 Hz—gamma), and one higher frequency band (215–230 Hz) that was assumed to only contain noise data. The dotted line shows the point at which the lower harmonic order model is 20 times less likely than the full cortical model. Note first that, in both inversion schemes, low order harmonic surfaces are very unlikely models for the data from the physiological (beta and gamma) frequency bands; indicating that these data likely derive from a grey matter structure. In contrast the noise data are supported equally well by all cortical models indicating that these data are unlikely to derive from the cortical surface.

For these data, recorded in a single subject, the functional estimates based upon MNM assumptions follow the anatomy more closely (i.e. they allow us to reject more of the lower order models) than those based on MSP assumptions for both physiological bands. We can now take the model set above the HDH and compute the posterior probability over these surfaces (Eq. (10)). In the MSP case (see S1 for MNM case) the posterior distribution (not directly shown here, but the integral over power change in Fig. 2C) peaks at cortical model *L* = 31 and this surface is shown in Fig. 2B with the *t* statistic map of the 15–30 Hz power change from 1 s before to 1 s after stimulus onset. We can now combine the posterior densities over cortical models and power change to give a joint probability at any cortical location. In Fig. 2C this joint probability distribution is shown for the location of peak modulation (white cross) in Fig. 2B. An ideal distribution would occupy only the highest harmonics (meaning that one could expect very low distortion). In this case (where cortical models below HDH have been assigned 0 probability) it is clear that there are a wide range of cortical models (from *L* = 15 to 30) that support a decrease in power at this vertex equally well. However around *L* = 10, increases and decreases in power are equally likely. Integrating over harmonic range and negative log power change allows us to say that there is a probability of 0.94 that there is a decrease in power at this vertex to within a distortion of ± 6 mm. Panel D shows this integral (for log modulation < 0 and *L* > 10) across the cortical sheet. Note that in both panels B and D we see the expected (Pfurtscheller and Lopes da Silva, 1999) contra-lateral modulation of the 15–30 Hz band within central sulcus consistent with right hand finger movement. Importantly however, this observation plays no part in our quantification of how accurate the images are.

To verify that this was not due to some characteristic of the data (i.e. white rather than coloured noise) or surface depth (see discussion) we also re-analysed the 30–60 Hz band data, but this time randomly permuted the channel lead-fields in order to destroy the geometrical relationship between the MEG data and the anatomy. Again, reassuringly, we found no difference between anatomical models at any spatial scale (diamonds on Fig. 2A).

Interestingly the relative accuracy of the two algorithms runs counter to our expectation that the MSP algorithm (which uses sparse patches) would improve over the MNM (in which no sparseness is enforced). We should also note that the absolute (rather than relative) model evidence for the MSP full cortical model (*L* = 42) was higher than MNM for both physiological bands (2.0 and 2.8 log units for 15–30 Hz and 30–60 Hz bands respectively). That is, although the MSP model was able to explain more data relative to its complexity, it was less sensitive to changes in cortical structure than the MNM model. The MSP model evidence also improved over MNM in the control conditions although the differences were smaller (0.07 and 0.8 log units for 215–30 Hz and permuted channel data respectively).

## Discussion

We have shown, for the first time, that it is possible to quantify the accuracy of a non-invasive functional brain image without recourse to the ground truth (which is almost never available). This is of direct relevance to all non-invasive brain imaging methods; *but importantly provides an objective function to differentiate between functional assumptions made by different MEG/EEG inverse solutions, for any dataset*.

In simulation, where data were generated in accordance with MSP assumptions, we saw the power of the technique to differentiate between inversion algorithms. In contrast, for the real data example, the two algorithms had similar performance. Importantly, one could use this objective and non-invasive metric of distortion to refine M/EEG inversion assumptions. These refinements not only include the appropriate prior assumptions to reflect cortical current flow (sparse or distributed in MSP and MNM respectively), as illustrated here, but could also include geometry-defining parameters such as surface vertex spacing and volume conductor models (Henson et al., 2009).

Here we compared an algorithm based on priors that consisted of a sparse set of patches of approximately 10 mm FWHM (MSP) with another based on a prior of uniform variance over all possible sources (MNM). For the simulated data, MSP priors performed better when the sources were simulated under MSP assumptions, whereas MSP and MNM priors performed similarly when the sources were simulated under MNM assumptions. One reason why MNM did not exceed the performance of MSP is that MSP has the capacity to reconstruct the MNM prior (or at least a smoothed version of it) through the recruitment of all patches. We were surprised however that for the real data we not only got approximately the same solutions with the two algorithms (compare Fig. 2 with supplemental S1), but that the MNM solution showed more sensitivity to the cortical structure (i.e. less distortion). Perhaps this is not surprising given a number of factors. Firstly, the sources of interest were predominantly superficial, at which level both algorithms have similar localizing performance (Friston et al., 2008b); secondly the localization was based on the ratio of source power differences, mitigating some of the inherent depth bias in the (MNM) scheme (similar to a dSPM (Dale et al., 2000)). Furthermore, we know MSP, which involves a high dimensional optimization, to be very sensitive to small coregistration errors (Lopez et al., 2012b); in contrast the MNM scheme needs simply to optimize a single regularization parameter. It maybe that the price paid for flexibility (in terms of optimization over priors) of the MSP scheme is that it is less robust to sensor and coregistration noise than MNM. By the same argument, even in the absence of coregistration noise, MSP is likely to be more sensitive to imperfections in the Nolte, 2003, forward model. We should also note that for the real data, MSP solutions had consistently greater evidence than MNM, it was just that the solutions were not as sensitive to changes in the cortical sheet—implying that MSP was explaining away variance due either to activity originating in sub-cortical structures (not modeled here) and/or external noise (note that MSP and MNM explained on average 96.0 and 96.3% of the data respectively). We should stress that this in no way constitutes a formal comparison of the two inversion algorithms (as it is based on a single subject) but simply outlines the method; further studies and many more subjects will be necessary to quantify the utility of different prior assumption sets.

Another important consideration is how the harmonic series will differentially affect different inversion assumptions such as those with bias towards superficial sources (like MNM) for example. As harmonic order decreases, the sulci and gyri become smoothed out leaving an ellipsoidal surface at mean cortical depth. As harmonic order (and hence the distance between the tops of gyri and depths of sulci) increases then any inherent depth bias will tend to polarise the current distribution towards either deep or more superficial regions. If the true current distribution is also polarised (and in the same direction as the inversion bias e.g. all superficial sources and MNM) then this could lead to apparent improvements in an algorithm with depth bias over one without. If this proved to be a problem in real data then one could consider using a different basis set (see below) in which all surfaces occupy the same depth range. Note however that one of the motivations for permuting channel labels was to verify that MNM solutions (Fig. 2, green diamonds) would not improve, regardless of the data, simply because of this increased depth modulation.

In this study we used a spherical harmonic basis set; an interesting avenue for further research would be to experiment with alternative basis sets. Earlier work (Barnes et al., 2006) looked at rotations of the grey matter volume, but tests had to be conducted within a spherical region of interest and no quantification of distortion was possible. One could explore the use of phase-randomized versions of this spherical harmonic set (to create distorted surfaces with the same spatial frequency content), or spherical wavelets (Yu et al., 2007). Another potentially interesting possibility would be to use basis sets derived from either the grey-CSF or grey-white cortical surface. The differences in curvature (coded in the harmonics) of these two surfaces in addition to their relative spatial displacement could also be a way to differentiate between sources in different cortical layers. Clearly the achievable resolution will be limited by coregistration error in practice, but we hope that this type of work will now become more tractable using headcasts (Troebinger et al., 2013).

The methodology introduced here is general and could equivalently be used to validate any result from non-invasive functional neuroimaging of the cortex. For instance, fMRI studies (van der Zwaag et al., 2009) have assessed the spatial accuracy of BOLD mapping across field strengths, with higher field BOLD responses having larger weighting towards microvasculature. In other studies (Harmer et al., 2012), the relative merits of spin echo (SE) and gradient echo (GE) planar imaging (EPI) for BOLD measurements have been probed, with spin echo theoretically giving better localization since static field inhomogeneities (i.e., around large veins) are refocused. The present methodology provides an unbiased robust statistical framework with which to answer such methodological questions; giving spatial confidence limits for non-invasive functional neuroimaging. Clinically for example one would be able to produce a posterior estimate of how the magnitude of an epileptogenic spike changes as the cortical surface model changes. Estimates that are more sensitive to distortions from the true cortical surface model (given that this is known) are likely to be more precise. From a general neuroscience perspective, it allows direct and quantifiable spatial comparison between invasive and non-invasive estimates of brain function across species.

## Appendix A Supplementary data

Fig. S1 The figure uses the same data as Fig. 2 from the manuscript but shows instead the minimum norm (MNM) specific estimates. For comparison the MSP solution from Fig. 2B is showing again in Panel A. Panel B shows the MNM estimated *t*-statistic map of power change (1 s pre vs. 1 s post stimulus) in 15–30 Hz band power for the cortical model (*L* = 42) with greatest probability from the fixed effects analysis (i.e. in this case the best model was the true model) on the candidate models above the HDH threshold (models 10–42 in this case). Panel C shows joint distribution over beta (15–30 Hz) band modulation (as a log of the power ratio so that negative values mean power decrease) and cortical model. As the range of harmonic surfaces from *L* = 11 to *L* = 42 support these data equally well (the curve is not strongly peaked at any harmonic) we can say that the spatial error bounds on this estimate are around ± 6 mm. In this case all viable cortical models show the same modulation estimate. In Panel D we show the integral, of probability of a distortion less than 6 mm (*L* > 10) and a power decrease, across the whole cortical surface.
