# Supplementary material for: Does function fit structure? A ground truth for non-invasive neuroimaging
Source: Neuroimage. 2014 Jul 1;94:89–95. doi: 10.1016/j.neuroimage.2014.02.033 (PMC4073649; doi:10.1016/j.neuroimage.2014.02.033)
Supplement: Fig. S1 — The figure uses the same data as Fig. 2 from the manuscript but shows instead the minimum norm (MNM) specific estimates. For comparison the MSP solution from Fig. 2B is showing again in Panel A. Panel B shows the MNM estimated t-statistic map of power change (1 s pre vs. 1 s post stimulus) in 15–30 Hz band power for the cortical model (L = 42) with greatest probability from the fixed effects analysis (i.e. in this case the best model was the true model) on the candidate models above the HDH threshold (models 10–42 in this case). Panel C shows joint distribution over beta (15–30 Hz) band modulation (as a log of the power ratio so that negative values mean power decrease) and cortical model. As the range of harmonic surfaces from L = 11 to L = 42 support these data equally well (the curve is not strongly peaked at any harmonic) we can say that the spatial error bounds on this estimate are around ± 6 mm. In this case all viable cortical models show the same modulation estimate. In Panel D we show the integral, of probability of a distortion less than 6 mm (L > 10) and a power decrease, across the whole cortical surface. [file mmc1.pdf]

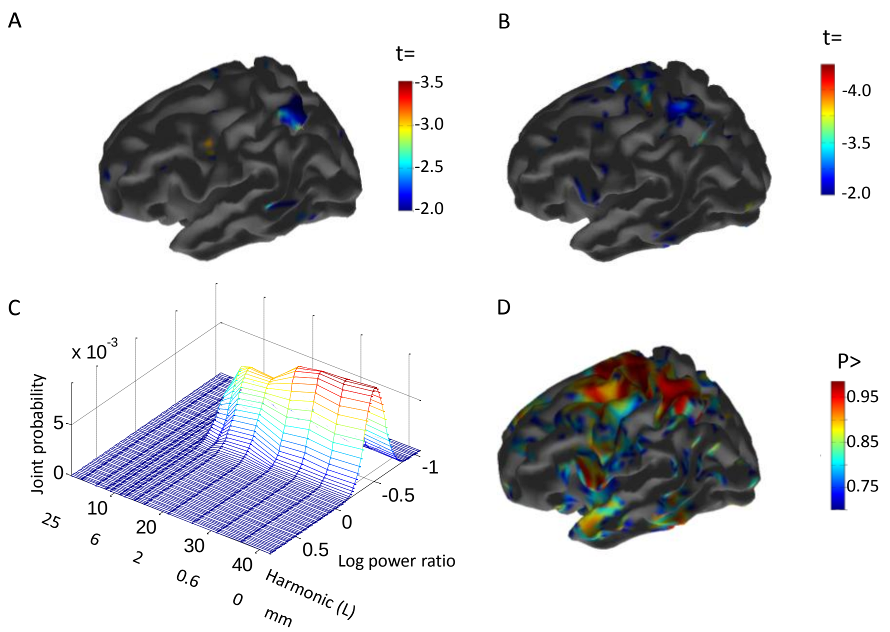

Figure S1. The figure uses the same data as figure 2 from the manuscript but shows instead the minimum norm (MNM) specific estimates. For comparison the MSP solution from figure 2B is showing again in Panel A. Panel B shows the MNM estimated t-statistic map of power change (1sec pre vs. 1 sec post stimulus) in 15-30Hz band power for the cortical model (L=42) with greatest probability from the fixed effects analysis(i.e. in this case the best model was the true model) on the candidate models above the HDH threshold (models 10-42 in this case). Panel C shows joint distribution over beta (15-30Hz) band modulation (as a log of the power ratio so that negative values mean power decrease) and cortical model. As the range of harmonic surfaces from L=11 to L=42 support these data equally well (the curve is not strongly peaked at any harmonic) we can say that the spatial error bounds on this estimate are around ±6mm. In this case all viable cortical models show the same modulation estimate. In Panel D we show the integral, of probability of a distortion less than 6mm (L>10) and a power decrease, across the whole cortical surface.
